# Evaluating the quality of research co-production: Research Quality Plus for Co-Production (RQ + 4 Co-Pro)

**DOI:** 10.1186/s12961-023-00990-y

**Published:** 2023-06-13

**Authors:** Robert K. D. McLean, Fred Carden, Alice B. Aiken, Rebecca Armstrong, Judy Bray, Christine E. Cassidy, Olivia Daub, Erica Di Ruggiero, Leslie A. Fierro, Michelle Gagnon, Alison M. Hutchinson, Roman Kislov, Anita Kothari, Sara Kreindler, Chris McCutcheon, Jessica Reszel, Gayle Scarrow, Ian D. Graham

**Affiliations:** 1 Faculty of Medicine and Health Sciences, Stellenbosch University, Tygerberg, South Africa; 2 Policy and Evaluation Division, International Development Research Centre, Ottawa, Canada; 3 Integrated Knowledge Translation Research Network, Ottawa Hospital Research Institute, Ottawa, Canada; 4 Using Evidence Inc., Ottawa, Canada; 5 Vice-President Research and Innovation, Dalhousie University, Halifax, Canada; 6 Research and Evaluation Branch, National Disability Insurance Agency, Melbourne, Australia; 7 Vice-President Research, Canadian Cancer Society, Toronto, Canada; 8 School of Nursing, Dalhousie University, Halifax, Canada; 9 School of Communication Sciences & Disorders, University of Western Ontario, London, Canada; 10 Dalla Lana School of Public Health, University of Toronto, Toronto, Canada; 11 Max Bell School of Public Policy, McGill University, Montreal, Canada; 12 Consultant, Ottawa, Canada; 13 School of Nursing and Midwifery, Centre for Quality and Patient Safety in the Institute for Health Transformation, Deakin University, Geelong, Australia; 14 Barwon Health, Geelong, Australia; 15 Faculty of Business and Law, Manchester Metropolitan University, Manchester, United Kingdom; 16 School of Health Sciences, The University of Manchester, Manchester, United Kingdom; 17 School of Health Studies, Western University, London, Canada; 18 Department of Community Health Sciences, University of Manitoba, Winnipeg, Canada; 19 George & Fay Yee Centre for Healthcare Innovation, University of Manitoba, Winnipeg, Canada; 20 Ottawa Hospital Research Institute, Ottawa, Canada; 21 School of Nursing, University of Ottawa, Ottawa, Canada; 22 Michael Smith Health Research B.C, Vancouver, Canada; 23 Clinical Epidemiology Program, Ottawa Hospital Research Institute & Schools of Epidemiology and Public Health & Nursing, University of Ottawa, Ottawa, Canada

**Keywords:** Research co-production, Research Quality Plus, Research Quality Plus for Co-Production, Integrated knowledge translation, Community based participatory research, Engaged scholarship, Participatory research, Research evaluation

## Abstract

**Background:**

Co-production is an umbrella term used to describe the process of generating knowledge through partnerships between researchers and those who will use or benefit from research. Multiple advantages of research co-production have been hypothesized, and in some cases documented, in both the academic and practice record. However, there are significant gaps in understanding how to evaluate the quality of co-production. This gap in rigorous evaluation undermines the potential of both co-production and co-producers.

**Methods:**

This research tests the relevance and utility of a novel evaluation framework: Research Quality Plus for Co-Production (RQ + 4 Co-Pro). Following a co-production approach ourselves, our team collaborated to develop study objectives, questions, analysis, and results sharing strategies. We used a dyadic field-test design to execute RQ + 4 Co-Pro evaluations amongst 18 independently recruited subject matter experts. We used standardized reporting templates and qualitative interviews to collect data from field-test participants, and thematic assessment and deliberative dialogue for analysis. Main limitations include that field-test participation included only health research projects and health researchers and this will limit perspective included in the study, and, that our own co-production team does not include all potential perspectives that may add value to this work.

**Results:**

The field test surfaced strong support for the relevance and utility of RQ + 4 Co-Pro as an evaluation approach and framework. Research participants shared opportunities for fine-tuning language and criteria within the prototype version, but also, for alternative uses and users of RQ + 4 Co-Pro. All research participants suggested RQ + 4 Co-Pro offered an opportunity for improving how co-production is evaluated and advanced. This facilitated our revision and publication herein of a field-tested RQ + 4 Co-Pro Framework and Assessment Instrument.

**Conclusion:**

Evaluation is necessary for understanding and improving co-production, and, for ensuring co-production delivers on its promise of better health.. RQ + 4 Co-Pro provides a practical evaluation approach and framework that we invite co-producers and stewards of co-production—including the funders, publishers, and universities who increasingly encourage socially relevant research—to study, adapt, and apply.

**Supplementary Information:**

The online version contains supplementary material available at 10.1186/s12961-023-00990-y.

## Background

### What is research co-production?

Research co-production is an approach to generating knowledge where researchers work in partnership with research beneficiaries and/or research users. Together, a co-production team aims to identify problems worth solving, design a research strategy that makes sense to all involved, interpret the meaning and merit of what is discovered for each party, and share and possibly implement findings collaboratively. In a recent book, the editors of *Research Coproduction in Healthcare* define research co-production as:

“… a model of collaborative research that explicitly responds to knowledge user needs in order to produce research findings that are useful, useable, and used.” [1]

We accept this definition and hereafter use ‘research co-production’ as an umbrella term. We do not carve sharp edges around what the term ought to mean. To the contrary, in this effort to develop a framework for evaluating co-production we aimed to build and test an approach that might be relevant across the many traditions of collaborative health research (the approach has been tested only with health research, but we anticipate it might be useful in other fields such as climate or agriculture sciences for example). In our previously published study protocol, we outlined how the Research Quality Plus for Co-Production (RQ + 4 Co-Pro) Framework presented herein may be applicable to collaborative research traditions  of Participatory Research, Integrated Knowledge Translation, Engaged Scholarship, Mode 2 Research, or Community-Academic Partnership (see Table 1 in McLean, Carden, Graham et al. [2]). Following the research we report here, we posit that RQ + 4 Co-Pro holds potential across and beyond these traditions, which in turn establishes an empirical question and opportunity for future users of RQ + 4 Co-Pro to test.

### The emerging potential of research co-production

Multiple benefits of research co-production have been hypothesized, claimed, and in some cases documented, in the academic and practice record. To help accurately track and categorize co-production benefits, Sibley et al. reviewed the peer-reviewed literature on co-production results and proposed seven categories of outcomes and impacts—what these authors call ‘effects’ [3]. These seven categories include: (1) effects on the research process (e.g., setting research priorities), (2) effects on relationships (e.g., trust in research), (3) effects on individuals (e.g., empowerment and confidence), (4) effects on research outputs (e.g., enhanced relevance), (5) effects on practice/programs (e.g., influence on service delivery), (6) effects on communities (e.g., community empowerment), and (7) effects on policies/systems (e.g., policy change). Although the authors note that the evidence base for many of these results is empirically weak and causal pathways remain unclear, there is a wealth of promising findings from each category, across settings, geographies, and domains of health sciences [3].

As a co-production team with significant experience outside academia, we note that a review of peer-reviewed literature provides a useful, but incomplete, picture of the full range of benefits and negative consequences of research co-production.

Concurrently, some research funders with interest in the approach are working to ensure the hypothesized benefits of co-production approaches are identified and demonstrated. For instance, in a large-scale evaluation of co-production research from across Canada, the Canadian Institutes of Health Research (CIHR) found co-production (referred to as integrated knowledge translation by CIHR) was: (1) more likely to influence the behavior of users including decision-makers in health-care settings, and, (2) more likely to contribute to real-world applications vis-a-vis a comparative counterfactual group of traditional researcher-only driven projects [4]. Globally, the same results are emerging. For one example, in a review of 200 completed research projects from across the world, the International Development Research Centre (IDRC) identified the engagement of stakeholders in and throughout the research process as an enabler of research impact achieving an optimal scale of benefit [5].

### Co-producing an evaluation framework for assessing the quality of research co-production

Two challenges justified our work to develop a framework for evaluating the quality of co-production. The first challenge is *instrumental*. For research co-production to achieve its presumptive potential, and for co-producers to be incentivised and rewarded for their work, co-production requires evaluations that are conducted in a rigorous manner and that provide meaningful insights for users. How can we as co-producers know what’s working well, and how can co-producers improve their craft without relevant and useful quality improvement tools? As James Lavery argues: “…to become more widely accepted by funders and researchers, and to contribute more conspicuously to the success of science programs and policy, it [community and stakeholder engagement in research] will have to establish a more coherent and convincing body of evidence…” [6].

The second challenge driving our effort is *methodological*. There is a notable gap in the practical options available for evaluating the quality of research co-production. Recent reviews of frameworks for implementing and managing research co-production indicate they have limited success in supporting the evaluation of co-production, and they do not contain the required scientific validation and grounding for systematic and trust-worthy evaluative application (7; 8). In our study protocol and a conceptual chapter, we reviewed and situated our effort within this literature—readers may be interested in reviewing this justification in McLean et al. [2] and McLean et al. [9].

To respond to these challenges, we embraced the spirit of co-production and undertook this study as a team of researchers and knowledge users/beneficiaries, for example members of our team are research funders, journal editors, educators, evaluators, and health system decision-makers from the public and not-for-profit sectors.

### RQ + and RQ + 4 Co-Pro

The Research Quality Plus for Co-Production (RQ + 4 Co-Pro) Framework and Assessment Instrument builds on the work of the International Development Research Centre (IDRC) and the Research Quality Plus (RQ +) approach. The International Development Research Centre is a Canadian research funder with offices in Ottawa, Amman, Dakar, Montevideo, Nairobi, and New Delhi. IDRC funds ‘research for development’, in other words, research that is intended to support human, social, and environmental prosperity. Dissatisfied with shortcomings in the mainstream methods of research evaluation, IDRC worked with its research and evaluation community to develop and implement the RQ + approach in order to support assessment of the quality of the use-oriented research it funds [10]. It has been used both in high income countries as well as low- and middle-income countries. The RQ + approach introduced three tenets for holistic research quality evaluation, and we use these tenets as our conceptual building blocks. These tenets include: (1) context matters for any evaluation of research, (2) research quality is a multi-dimensional, values-driven concept, and (3) evaluations of research must be empirical and systematic, not only based on peer opinion [10]. RQ + was first published by IDRC following a practical application across a series of 170 research project evaluations [11]. In 2022, IDRC updated RQ + following its 2020–21 application in another (different) series of 160 research project evaluations [12]. Today, RQ + maintains the three tenets and provides a validated, alternative vision for defining and assessing research quality [12]. It should be clear that the focus is on research quality, not outcomes of the research.

Our RQ + 4 Co-Pro Framework and Assessment Instrument tailor the IDRC’s RQ + approach for the specifics of research co-production. The RQ + 4 Co-Pro Framework (presented later in this paper as Fig. 2) outlines this versioning, including updates following our field-test and iterative co-development process. It was originally developed by authors RKDM, IDG, and FC as a conceptual framework and published as a prototype in McLean et al. [9]. This manuscript presents how that conceptual version was field-tested and revised via team deliberation of the pilot test study results. Additional file 1 presents the field-tested and co-developed RQ + 4 Co-Pro Assessment Instrument—a practical tool that evaluators might use (in this field-test they did) to operationalize the Framework in an evaluation of co-production. To our knowledge, this study represents the first adaptation of IDRC’s RQ + approach to the specifics of a particular research field, approach, or methodology.

### Study objectives & research questions

The objective of our research was to test the relevance and utility of the novel RQ + 4 Co-Pro Framework. Two research questions guided the work:

- 1. Is the RQ + 4 Co-Pro Framework relevant for the evaluation of research co-production?
- 2. Is the RQ + 4 Co-Pro Framework useful for the evaluation of research co-production?

Box 1. The RQ + approach, The RQ + 4 Co-Pro Framework, and RQ + 4 Co-Pro Assessment Instrument **The Research Quality Plus (RQ +) approach**

RQ + is a holistic approach to defining, managing and evaluating the quality of research. It is underpinned by three tenets: (1) Context matters to any evaluation of research; (2) Research quality is a multidimensional construct; (3) Like quality research, evaluations of research quality should be empirical not only peer-opinion based. An underlying principle of RQ + that is further explained in the instrument is that applied research and use-oriented basic research should be positioned for use. That is the research should be designed, carried out and delivered in a manner which is both clear and useful to the primary users and beneficiaries.

**The Research Quality Plus for Co-Production (RQ + 4 Co-Pro) Framework**

Embracing the three tenets of the RQ + approach, the RQ + 4 Co-Pro Framework defines contextual factors, quality dimensions, and an empirical process for assessing the quality of research co-production specifically. Together these elements construct a purpose-built yet user-adaptable framework for conceptualizing and assessing co-production quality. See Fig. 2.

**The Research Quality Plus for Co-Production (RQ + 4 Co-Pro) Assessment Instrument**

The RQ + 4 Co-Pro Assessment Instrument (see Additional file 1) operationalizes the RQ + 4 Co-Pro Framework. It provides instructions for users and defined evaluative rubrics for each Framework component. The Assessment Instrument facilitates an empirical and systematic evaluation of contextual factors and quality dimensions associated with co-production.

## Methods

Our co-production team published a study protocol in open access [2]. In this section we outline this methodological approach and highlight any variance from the protocol. We used the Standards for Reporting Qualitative Research (SRQR) reporting checklist, see Additional file 2 [13].

### Co-production approach & team positionality

Following a co-production approach, the research was undertaken by a team of researchers and research beneficiaries from a broad range of professional backgrounds. All members participated through the lifespan of the research process, collaborating to develop study objectives, questions, methodological design and results sharing strategies. This collaboration was conducted using virtual MS Teams meetings and email-based document exchange. Although the project employed a collaborative approach to all steps, within the team authors RKDM, FC, and IDG were primarily responsible for oversight of research implementation and led activities such as data collection and academic reporting. Authors ABA, RA, JB, CEC, OD, EDR, LAF, MG, AMH, RK, AK, SK, CM, JR, GS were primarily responsible for providing beneficiary/user perspectives through research design and implementation and identifying opportunities for results to be shared and applied. Purposefully, the co-production team was constructed to represent critical user, beneficiary, and gate-keeper authorities for research co-production, including co-production scientists, journal editors, university leadership and administration, graduate students, funders, research network management, research evaluators, public policy makers, health systems consultants, and not-for-profit/foundation senior leadership. We acknowledge our team is not exhaustive and does not represent the views of all potential users or beneficiaries of better co-production evaluation (for one pertinent example: patients). Our team was constructed to bring together professional perspectives, that we believed, would be in a position to first use a tested co-production evaluation approach. The co-production team included members based in Australia, Canada, and the United Kingdom. Members of the team self-reported gender, and the aggregate profile is 13 women (72%), 5 men (28%) and 0 non-binary (0%).

### Research design

**Fig. 1:**
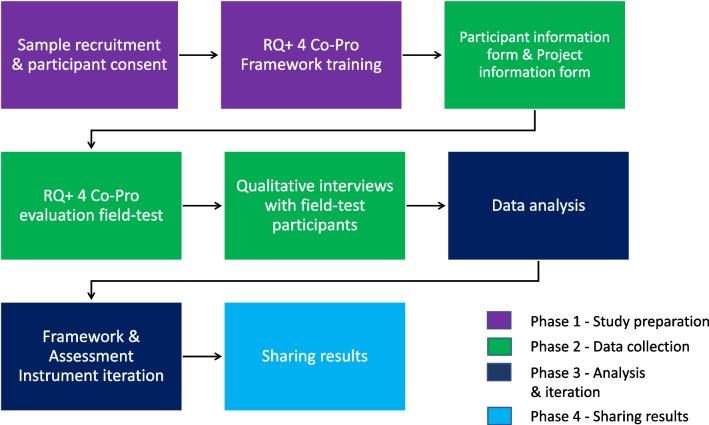
Research life cycle

We used a constructivist paradigm (wherein those involved construct their own knowledge of the world through experience and reflection) and employed a multiple methods qualitative design [14, 15]. The constructivist approach facilitated co-production across perspectives on our team, by centering the importance of deliberating varied experience and interpretation in our practice and research process [16]. The constructivist approach supported qualitative data collection by elevating participants’ conceptual views about and experiences with the RQ + 4 Co-Pro field-test, and the paradigm of constructivism drove our use of an inductive approach to data analysis [16, 17]. We used standardized self-reported participant and project templates, training of participants, participant-led dyadic evaluations of projects, and follow-up qualitative interviews with participants (who were all both assessors and those whose projects had been assessed). To develop and revise the RQ + 4 Co-Pro Framework, the co-production team employed a process of collective examination of empirical results and deliberative dialogue, an approach to supporting people and communities to engage in dialogue with each other [18–20]. RKDM and FC interviewed the assessors individually following submission of the assessments to understand how the process worked for them and to identify issues each felt needed to be addressed. RKDM and FC consolidated this information and proposed revisions to the team for their input and further reflections. Based on input from all team members, RKDM and FC made further revisions to prepare the final versions. This process allowed the team to identify possible revisions and select desirable changes by a process of creating consensus [16]. Figure 1 illustrates the life cycle of the research.

### Phase 1—study preparation

#### Sampling

Individual participants and individual projects were purposefully drawn from the Integrated Knowledge Translation Research Network (IKTRN). The IKTRN is an international network of researchers and knowledge users who practise and study integrated knowledge translation [21]. We identified IKTRN as the sample universe on two grounds. First, to ensure study participants were suitably skilled and experienced in co-production. Second, members of the IKTRN would be able to submit a recent co-production project of their own for dyadic evaluation. Sixteen to 20 participants were identified by our co-production team as an estimated sample size that would lead to saturation [2, 22]. We were open to increasing the sample size if the saturation estimate did not hold (in terms of data richness). As discussed under the interview analysis section, this original sample size estimate in fact did hold. The IKTRN Director recruited network members through email correspondence. Once a sample of 20 individuals was recruited, we accepted all 20 participants and obtained informed consent for study participation. Recruited individuals were asked to identify a recent IKT study with which they were involved. No other limitations were placed on the identified study. During the study, 2 participants dropped-out due to competing work demands, leaving 18 active participants. We believe it brought strength to the study to recruit experienced co-production specialists. We note, however, this expertise and experience will be reflected in the findings and how they should be interpreted. We may not have reached the same findings with a sample of researchers who do not regularly practice co-production.

#### RQ + 4 Co-Pro training of participants

We hosted an online training session that introduced study participants to the RQ + approach and the novel RQ + 4 Co-Pro Framework we developed in prototype version for this study. The training session was two hours in length. It was led by members of the co-production team with extensive experience using the RQ + approach at the International Development Research Centre and at the Global Challenges Research Fund (RKDM & FC).

### Phase 2—data collection

#### Participant & project information forms

Each study participant completed two digital forms. The participant information form collected basic demographic details about the participant, including gender, years of experience with co-production, and years of experience in research. The project information form elicited basic information about the identified project the participant would represent in the evaluation simulation, such as project length, funding amount, and type of knowledge-users involved. Collecting systematic data about each participant and project allowed us to examine and better understand the evaluators and evaluands (subjects of evaluation), that made-up the field-test.

#### RQ + 4 Co-Pro evaluation field-test

Following training and basic information collection, study participants were randomly assigned into dyadic sets. We launched with ten dyads, but as noted earlier, one pair dropped out of the study leaving 18 participants in nine dyads. Dyads were the core structure of the RQ + 4 Co-Pro field-test [23]. Dyads exchanged project background publications and scheduled their own interviews. Using data collected from project documents and the interview, each participant used the RQ + 4 Co-Pro Assessment Instrument to evaluate their partner’s project. The prototype Assessment Instrument used by study participants was published as Additional file 1 in our study protocol; see: McLean et al. [2]. The field-tested version, including updates and changes driven by this research, is described in the following section of this paper and published as Additional File 1. Completing the field-test required participants to evaluate their dyadic partner’s project that included all three tenets of the RQ + Approach: (1) considering context, (2) reviewing and assessing multiple dimensions of quality, (3) use of an empirical and systematic approach that incorporated a variety of data sources to triangulate findings. Dyads did not return their completed RQ + 4 Co-Pro Assessment Instruments to the research team as the purpose of our research was not to assess the quality of the sampled projects but to test the relevance and utility of the RQ + 4 Co-Pro Framework.

#### Qualitative interviews with study participants

To learn about participants’ experiences using the RQ + 4 Co-Pro Framework and Assessment Instrument in the field-test, we interviewed each study participant independently. We elected to use a qualitative approach to data collection to capture the context, diversity and richness of experience within the participant sample. Each qualitative interview used a common guide but was approached in a semi-structured manner to capture the feedback each independent interviewee found to be most pertinent to their experience. Interviewers (RKDM, FC) conducted the first interview together to ensure consistency of the approach and debriefed on the experience to discern possible improvements. Thereafter, interviews were conducted independently. The interviewers exchanged notes as interviews were completed to enhance coherence in the approach and ensure emergent learning was built into both interviewers’ work. Interviews were conducted using the MS Teams platform and lasted between 45 and 90 min. Interviews were audio recorded and transcription was completed by the interviewer. Alternatively, with permission of the interviewee, transcriptions were generated within the MS Teams platform in real time and the interviewer took notes concurrently to censure a complete record of the interview was captured.

### Phase 3—analysis & iteration

#### Data analysis

All data for each participant (transcripts and notes from interviews, reflections with participants, project information forms, participant information forms) were assigned a random number identifier for confidentiality purposes.

Project and participant information forms were analyzed using frequencies for close-ended questions. Open-ended questions were reviewed for common or disparate themes [24].

To analyze interview data, we used thematic analysis to identify patterns in the interview data [25]. We used an inductive, or data-driven approach, without using a pre-existing coding frame. As interviews were completed, the interviewers (RKDM, FC) met to discuss emerging themes and experiences to iteratively develop a data coding structure. These iteration meetings centered on cross-checking themes that were identified independently by the two lead analysts (RKDM, FC), and continued until all interviews were complete and consensus between the analysts was reached. Next, the coding frame for the interviews was agreed/completed in a full team meeting at the conclusion of all interviews. This allowed both a validity check and opportunity for insight from potential RQ + 4 Co-Pro users/beneficiaries.

We analysed data from each method (project information forms, participant information forms, qualitative interviews) separately using the above-described processes. Following independent analysis, we conducted triangulation across methods to identify patterns in the data. Our triangulation process was done through stratification of interview data by response categories in the project and participant information forms. We conducted stratified analysis for grant length, funder type, and participant experience teaching and supervising co-production. However, we identified no dominant patterns in the data when interview findings were stratified by project or participant characteristics. That is to say, the analysis produced comparable results under each stratified analysis. Consequently, we report study results in aggregate and by source/method in the following section of this paper.

#### Framework and assessment instrument iteration

**Fig. 2:**
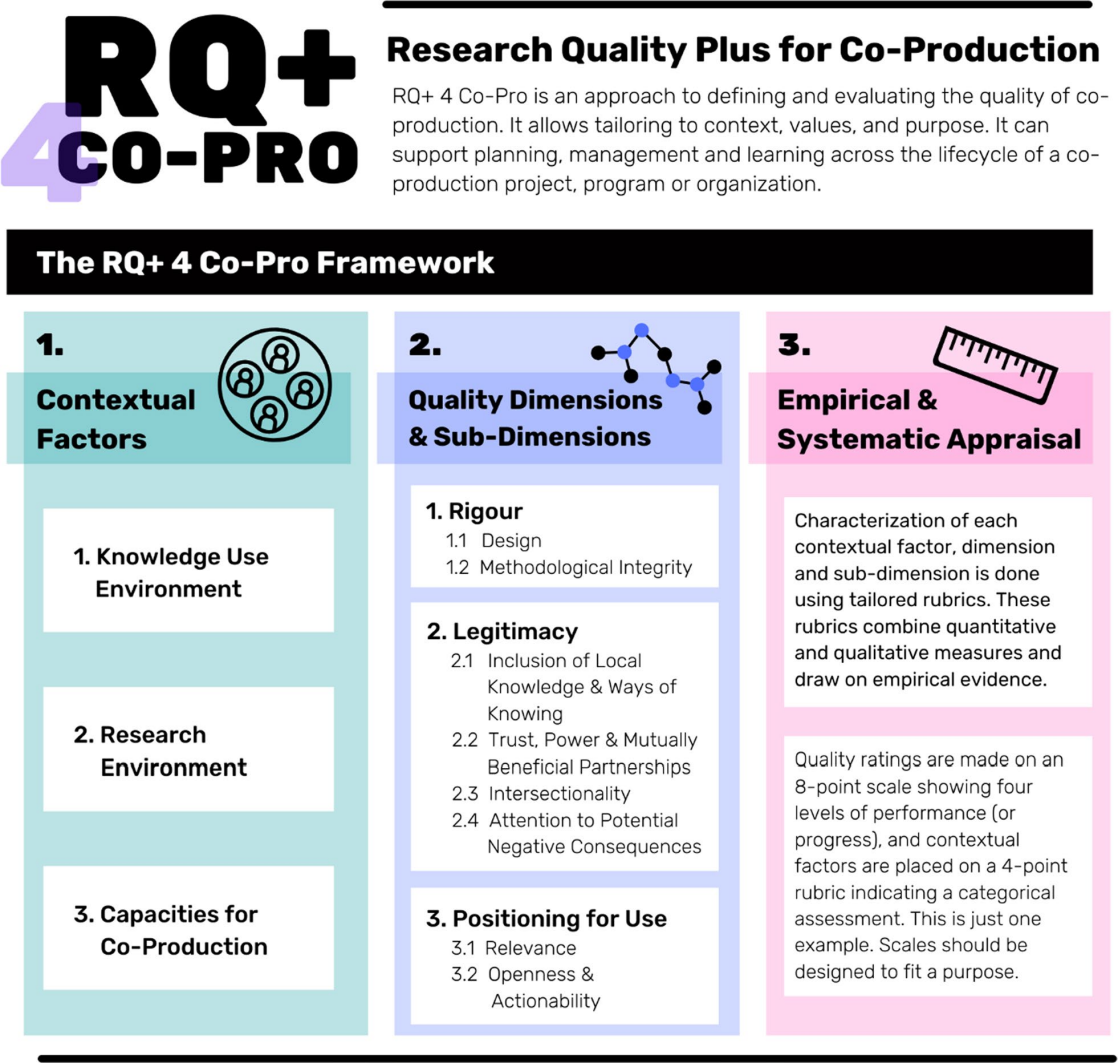
Presents the field-tested and co-produced RQ + 4 Co-Pro framework

Based on the data analysis, we revised the prototype RQ + 4 Co-Pro Framework and Assessment Instrument using deliberative discussion as a co-production team [18–20]. To facilitate the deliberative revision process, we held two meetings of the co-production team where we discussed findings and recommendations derived from the interviews. Changes were agreed to by consensus at each meeting. In addition to team meetings, we coordinated deliberation and revisions via email; the final iteration was approved by all team members and is presented as Fig. 2 (the RQ + 4 Co-Pro Framework) and Additional file 1 (the RQ + 4 Co-Pro Assessment Instrument). In alignment with our research questions, we used two criteria to identify possible changes: (1) relevance of RQ + 4 Co-Pro for research co-production, and, (2) utility of RQ + 4 Co-Pro for co-production evaluation.

### Research setting

All research activities were undertaken online making use of virtual communications and data sharing technologies as described above. All data was stored on a secure drive of the Ottawa Hospital Research Institute.

### Research ethics

Research ethics approval was provided by the Research Ethics Board of the Ottawa Health Science Network (OHSN-REB 20210642-01H).

## Results

### Participant characteristics

Summarizing results of the participant information forms, Table 1 provides an overview of the co-production specialists who participated in the RQ + 4 Co-Pro field-test. Overall, participants held significant experience in doing, teaching, and supervising research co-production. All participants self-reported their gender as woman.

**Table 1:** Profile of participant sample for RQ + 4 Co-Pro field test

	Gender			Highest degree held			Supervisory experience		# of years research co-production experience			# of co-production projects completed			Teach co-production	
*n*	Woman	Man	Non-binary	Doctorate	Masters	Bachelors	Yes	No	Mean	Median	Mode	Mean	Median	Mode	Yes	No
18	18	0	0	16	1	1	11	7	9	9.7	5	2.5	5.5	2	12	6

### Project characteristics

Summarizing results of the project information forms, Tables 2 and 3 provide an overview of the research co-production projects included in the RQ + 4 Co-Pro field-test. All projects utilized mixed methods approaches in their design. Projects addressed a broad range of health issues from indigenous health to oncology to COVID-19, and engaged a diverse range of research beneficiaries (see Table 2). On average, projects included nearly twice as many research beneficiary team members (13.8) than researcher team members (7.4) and lasted just under 4 years (47 months).

**Table 2:** Profile of projects assessed in the RQ + 4 Co-Pro field-test

Domain summary	Dates active (mo/yr)	Length of project (months)	# of researchers involved (including trainee team members)	# of research beneficiaries (by type)	Country where research was housed
Health systems	04/20–04/23	36	7 including 1 Postdoc, 1 PhD	PM = 2 HCS Admin = 2 NFP Admin = 3 Clinician = 4 Educators = 4 Patients = 2	Canada
Pediatrics	03/15–01/18	34	5 including 1 PhD	HCS Admin = 1 Clinician = 2	Canada
Pediatrics	04/17–09/19	30	3 including 1 Master’s	HCS Admin = 2 Clinician = 4 Family = 1	Canada
IKT for health care providers	10/14–03/19	54	3 including 1 PhD	Clinicians = 5	Ireland
Maternal newborn care	09/13–09/18	60	14 including 2 PhD, 1 Master’s	PM = 2 Funder = 2 HCS Admin = 3 Clinician = 7	Canada
Health research training guide	09/17–02/21	42	20 including 2 Postdoc, 5 PhD	Funders = 4 NFP Admin = 5 Clinician = 3 Lived experience = 6 Industry = 2	Canada & USA
Cancer & First Nations	10/17–10/19	24	8 including 1 Postdoc, 3 PhD, 1 Master’s	HCS Admin = 2 Clinician = 1 Educator = 1	Canada
Cancer & Inuit care	09/17–09/19	24	2	PM = 2 HCS admin + 4 NFP admin = 3 Clinician = 14 Educator = 2	Canada
Support to self-management in older adults with multi-morbidity	01/16–02/22	85	10 including 1 Postdoc, 1 Master’s, 1 Undergrad	PM = 1 Funder = 1 Clinicians = 10 Patients = 10 Tech developers = 3	Canada
Health system Advisory Councils	2018–2020	24	4	NFP admin = 5	Canada
Multi-jurisdictional health networks	08/13–08/21	96	16 including 1 PhD, 1 Master’s	PM = 2 HCSadmin = 34	Canada
Decision aid in cardiovascular disease	04/15–03/17	24	4 including 1 PhD	HCSadmin = 1 Clinician = 1 Patient = 2 Family = 15	Canada
Disability	06/15–05/16	12	5 including 3 Undergrad	13 including students, researchers, faculty and community	Canada
Humanitarian	12/16–12/19	36	4 including 1 PhD, 1 Undergrad	NFPadmin = 4 Clinician = 2	Haiti
Stroke survivors	01/19–07/19	7	13 including 1 Postdoc, 1 PhD	Clinician = 4 Patient = 1	Australia
Birthing centres/MNC	01/13–12/20	96	11	PM = 2 Funder = 5 HCSadmin = 7 Clinician = 6 Patient = 2	Canada
Oncology	2007–2019	144	18 including 1 Postdoc, 4 PhD, 1 Master’s	PM = 6 Funder = 1 HCSadmin = 4 Clinician = 15 Educator = 5 Patient = 3 Family = 1	Canada and USA
Rural & Covid-19 impacts	12/19–12/20	12	3 including 1 Postdoc, 1 undergrad	HCSadmin = 1 Clinician = 1	Canada

*Cdn* Canadian, *MNC* Maternal and Neonatal Care, *HCS* Health Care System Administrator**,** *NFP* Not-for-Profit Administrator, *IKT* Integrated Knowledge Translation, *PM* Policy Maker, *IKTRN* Integrated Knowledge Translation Research Network, *KT* Knowledge Translation

**Table 3:** Descriptive statistics for projects assessed in the RQ + 4 Co-Pro field test

	Length of project	# of researchers	# of research beneficiaries
Mean	47 months	7.4	13.83
Median	35 months	6	10
Mode	24 months	4	5

### Qualitative interviews

We report findings from interviews in four themes: (1) most common observations, (2) relevance, (3) utility, (4) uses. Under the first, we describe general findings of importance to the RQ + 4 Co-Pro Framework revision and the potential value of the Framework. The second and third theme relate directly to our guiding research questions. The final theme that emerged relates to potential uses of RQ + 4 Co-Pro.

We use a consistent scale to communicate results, where: “all participants” = 18; “a majority of participants” = 10–17; “half of participants” = 9; “a minority of participants” = 2—8; “one participant” = 1. We encourage readers not to place substantive weight on these quantifications of the qualitative data, but, we report using this structure to support interpretation and to provide a sense of the homogeneity/diversity of perspectives offered by participants.

### Theme one—common observations

“[Assessing research quality using RQ + 4 Co-Pro is] Good for the science, good for my practice.”—Interviewee (I)17

There was a unanimous (18/18) response from participants that the RQ + 4 Co-Pro Framework was an important and timely development in the realm of co-production research. Participants elaborated that the importance of the work was rooted in its novelty, as there are few practical evaluation tools for co-producers and co-production teams. Timeliness was related to novelty and practicality, but further elaboration by participants emphasized the increased need for critical evaluation approaches given the mounting belief that co-production can offer benefits for science and health outcomes [26–28]. To this end, all participants suggested RQ + 4 Co-Pro contributes to filling a major gap in co-production science.

The majority of participants expressed a personal benefit from participating in the study, which related to the use of the Framework in the dyadic evaluation process. Specifically, this involved reflecting upon the project experience they had across the multiple dimensions of the Framework as well as acquiring insights (in writing or verbally) from a co-production peer about how they approached the quality dimensions and navigated context in their project.

### Theme two—relevance

“This tool starts to build the empirical evidence behind partnership research.” (I9)

All participants endorsed RQ + 4 Co-Pro as relevant for a broad range of research co-production projects.

Participants expressed appreciation for the contextual factors embedded in the Framework. They argued the review of context was critical to fully understand any co-production process and noted that this factor is not included in the existing methods for evaluating research (not only co-production research). Some participants suggested new contextual factors could be included depending on the intent of the evaluation, and suggestions included: (1) size of the project (as indicated by the amount of funding received and duration of project implementation), (2) success of the project (i.e., did it create knowledge that was used?), (3) experience of the partnership (i.e., how did members of the co-production effort feel about working with each other?).

The tenet of multi-dimensional quality expressed in the Framework was appreciated by all participants. The existing quality dimensions in the RQ + 4 Co-Pro Framework were endorsed by the majority of participants with some suggestions for change, all of which fell under sub-dimensions of Legitimacy. Most discussed by participants was the sub-dimension “Intersectionality”. In the case of intersectionality, most participants noted that intersectionality was not in common use when their research was designed. Study participants were interested in applying it to their work but requested further clarification of how it should be interpreted and applied; a minority of participants suggested it may not be understood well enough to be applied systematically; one participant suggested it should be removed from the Framework altogether. The sub-dimension of “Attention to potentially negative consequences” was also discussed by a minority of participants. Some suggested that a notion of ‘unexpected’ and ‘positive consequences’ might be included. One participant raised the important question of whether or not “potentially negative consequences” included inconvenient research findings. This participant re-iterated this is a particular risk in research co-production, where findings may actually undermine the wants or needs of the knowledge user. For instance, where the findings challenge the preferences or established routines/norms of the research beneficiary.

Finally, although all participants suggested the RQ + 4 Co-Pro Framework was broadly relevant, a majority of participants suggested the language in the Assessment Instrument required some revision to ensure the voice of researchers and research beneficiaries were equally weighed both in the component descriptions and corresponding evaluative rubrics. These participants argued that the Assessment Instrument worked well for them as researchers, but the language might be inaccessible to their research beneficiary partners, and as a result application of RQ + 4 Co-Pro would be dominated by researchers and scientists.

### Theme three—utility

“I would love to use this tool for a brainstorm with partners to ask, how are we doing? Am I missing something that is important to you as knowledge users? (I5)

All participants reported the Framework was useful for the evaluation of research co-production. On average it took 3.91 h to complete the dyadic evaluation (review of partner’s project publications and background documentation + the dyadic interview + recording of results in the Assessment Instrument). A minority of participants suggested this evaluation required a greater degree of effort and intellectual investment when compared to previous research evaluations they had completed; at the same time, the same participants indicated that they appreciated the opportunity for reflection and felt that it was worth the time and effort.

All participants reported that the dyad interview (a part of the field-test) was essential to completing the assessment. A majority of participants reported that without the primary data collection they would not have been able to assess the Legitimacy quality dimension or the Contextual Factors sufficiently. This same interviewee reported that these components of the Framework—Legitimacy and Contextual Factors—were essential to a complete an accurate evaluation of research co-production.

One participant reported the use of primary data (the dyad interview) in this field-test caused significant self-reflection on the peer-review they had previously (and regularly) provided for journals and funding applications. This participant suggested the reliance of these previous reviews on strictly secondary data sources was concerning, given the enriching value provided by the primary data in the RQ + 4 Co-Pro approach.

A majority of participants suggested the Framework would work well in project reflections. In essence, this could be done by removing the evaluative rubrics and using questions or simply descriptive text to record conclusions or feedback about a project. A minority of participants suggested it would be preferable if the first uses of RQ + 4 Co-Pro were more descriptive and developmental than evaluative.

“…parts of the Framework were difficult to gather data on because these are not commonly reported or discussed in the papers or documentation. But this does not mean they aren’t important, even essential, to how we do co-production.” (I6).

### Theme four—uses of the framework

“It was fantastic in terms of self-reflection.” (I4)

All participants expressed strong support for future use of the RQ + 4 Co-Pro Framework. A minority reported they were already using it in their work designing a new co-production project or to reflect on a current project. A minority elaborated that uptake and use of RQ + 4 Co-Pro—in particular the Legitimacy dimension—would help to combat the persistent and damaging problem of tokenism in co-production. These participants suggested the holistic vision of quality and context would shed light on exploitative practices and/or poorly formed and likely ineffective or inefficient partnerships.

Many new ideas for uses of RQ + 4 Co-Pro were suggested by study participants. Three main types of other uses were identified:

1. Instrumental uses
  - As a co-production project or program design tool
  - As the basis for research co-production reporting guidelines
  - In funder criteria for co-production calls for proposals & proposal review
2. Developmental uses
  - As a relationship management/monitoring tool
  - For team building and expectation-setting
  - For universities to “walk the talk” of social purpose
  - As a self-assessment tool
3. Educational uses
  - For capacity strengthening (with students or new/current co-producers)
  - To structure a book or case book on principles of co-production

A majority of participants posited it will be essential to specify the object of evaluation prior to implementation clearly in any future application. That is to say, there needs to be clarity about whether the Framework is used for the evaluation of a co-production project, program, organization, network, paper, faculty, etc. These participants raised the metaphor of comparing ‘apples to apples’.

### Iteration of the RQ + 4 Co-pro framework and assessment instrument following data collection and analysis

Following analysis of the field-test findings, we met as a co-production team to discuss key findings and their implications for the RQ + 4 Co-Pro Framework and Assessment Instrument. Possible revisions were deliberated on by the team and drew on team members’ co-production knowledge, experience, and professional backgrounds. The aim was to reach the most relevant and useful iteration of RQ + 4 Co-Pro we could, as a team. This version is not static. Our collective experience indicates it should be treated as a dynamic tool—one that is tailored and re-imagined by new users and for new uses.

Figure 2 below presents the revised RQ+ 4 Co-Pro Framework. Additional file 1 presents the revised RQ + 4 Co-Pro Assessment Instrument. We have made changes to both the Framework and Assessment Instrument, ensuring all components are aligned. We updated the language in the descriptions of each component to further clarify the Intersectionality and Attention to Potentially Negative Consequences dimensions. We revised the language in the Scientific Rigour Dimension and Sub-dimensions to reflect a broader and more open understanding of knowledge creation. We reviewed each Contextual Factor and Quality Dimension and Sub-dimension description and rubric to ensure the perspective of the research beneficiary partner was evident. Changes were made to language in the rubrics to improve or simplify interpretability based on interview feedback. A comparison of changes can be drawn by accessing the prototype Assessment Instrument published in our concept chapter and study protocol [2, 9].

## Discussion

This research has demonstrated how the original three tenets of the RQ + approach [10, 12] can be re-imagined and re-cast in the context of research co-production. Participants in our study highlighted the particular importance of the three RQ + tenets (1-context matters, 2-quality as multi-dimensional, 3-systematic and empirical appraisal) in their dyadic evaluations. Moreover, participants also revealed how the tenets presented important opportunities for disrupting the status quo in order to improve user engagement in co-production research and how it will help the field to move forward scientifically and socially. Moreover, these results provide an important contribution to IDRC’s call to action for iterations of RQ + [12].

On average it took 3.91 h to complete the dyadic evaluation (review of partner’s project publications and background documentation + the dyadic interview + recording of results in the Assessment Instrument). A minority of participants expressed RQ + 4 Co-Pro required more effort and intellectual engagement than typical peer-reviews, but, these participants categorically suggested the added effort increased the quality of their evaluation.

As Wilson and Kislov note, a new generation of measures is needed to capture the uptake of knowledge, skills, and practices in implementation science. Our research has demonstrated that RQ + 4 Co-Pro is well positioned to fill significant gaps in implementation science theory, the practice of research co-production, and the evaluation of research co-production. Strong RQ + 4 Co-Pro assessments will require that the assessors have good knowledge of co-production research, and preferably good knowledge of the area of study.

We identify several ways RQ + 4 Co-Pro responds to leading calls for improving co-production evaluation. Firstly, RQ + 4 Co-Pro with its focus on Contextual Factors responds directly to the work of Kreindler who argued co-production evaluation should include measures of context alongside measures of outcomes [30]. Our empirical results reaffirm Kreindler’s argument and suggest RQ + 4 Co-Pro is positioned to take on this challenge in a novel way. Secondly, RQ + 4 Co-Pro has been built on the work of Ward and colleagues who make the case for equity holding a central position in the evaluation of co-production [31]. To these ends, RQ + 4 Co-Pro holds a specific Legitimacy quality dimension which embeds measures of equity in its sub-dimensions (trust, importance of local knowledge, intersectionality, and attention to potentially negative consequences of the research and its results), and holds equal weight to all other quality dimensions, including Rigour. We believe this will help to centre equity and intersectionality as integral and equally important values for co-production success. Thirdly, RQ + 4 Co-Pro has responded to the call of Russel et al. to ensure evaluations of co-production take into account rationales for stakeholder involvement, are clear about power dynamics, and give attention to the fact there may be negative consequences [32]. RQ + 4 Co-Pro has responded with specific measures—now practically demonstrated via this research—for sharing power and for tracking potentially negative consequences. Finally, Boivin et al. raise pertinent remarks in their systematic review of co-production evaluation tools, that such tools must be developed via scientific testing processes and that the tools should be themselves co-produced [7]. Our RQ + 4 Co-Pro field-test design and our co-production approach have embraced both recommendations. We agree they have strengthened our results. Boivin and colleagues also argue co-production evaluation tools must be more accessible (i.e., understandable and readable). Moving forward, we will work to ensure RQ + 4 Co-Pro makes its way into purpose-oriented formats (more is written on this in our first recommendation).

In our view, responding to these colleagues’ challenges with RQ + 4 Co-Pro raises the bar for what counts as “good” in research co-production. Some may argue this creates another challenging hurdle for co-production. Our view—informed by this research and our own practical experience—is these innovations in how we conduct quality assessment will help to steer the field toward a more holistic understanding of the effects of co-production work [3].

Informed by our research, we provide three recommendations for moving RQ + 4 Co-Pro forward into theory and practice:

### New uses and users of RQ + 4 Co-Pro should be considered

Our research findings indicate the potential for RQ + 4 Co-Pro to stretch beyond project evaluation. Although the evaluand in our field-test was a co-production research project, many participants argued there would be value in applying it to the study of alternative collaborative endeavors. This implies both new uses, and new users. For example, the evaluation of a manuscript reporting results of a co-production project by a journal editor or as a systematic guide for its peer-reviewers, the evaluation of an organization specializing in co-production by its administrator or its beneficiary community, or the evaluation of a program of co-production grants by a research funder. Many alternatives were suggested. For alternative uses to be realized we believe active sharing and socialization of study results (i.e., making them a part of standard co-production research assessment), will be required beyond academic journal publication. As a co-production team we will start by considering the potential for educational uses of RQ + 4 Co-Pro (e.g., in the research curriculum), as well as promoting it in a range of fora to stimulate interest and alternative uses of the instrument.

### Use RQ + 4 Co-Pro before, during and after co-production

Study participants argued RQ + 4 Co-Pro should not be limited to post-hoc evaluation. We agree and, based on data collected from the dyads, identify potential for using the Framework before, during and after a co-production process.

***Before*** a project, the Framework could be applied as a design tool helping to lay out shared expectations between members of a team, or as a guide to draft a co-production proposal. In the same way, a funder could use the Framework to assess co-production project proposals, or provide it as guidance to its peer review committees.

***During*** a project, RQ + 4 Co-Pro could help to monitor context and elements of quality important to the project, introducing modifications into the chosen coproduction approach as and when required. As the project team advances the work, they could use the tool to raise discussions across team members about progress and evolving expectations against the Framework components.

***After*** co-production, RQ + 4 Co-Pro could be used as a post-hoc tool and in ways that stretch beyond project evaluation employed in this study. For example, it could be used for communicating results to researcher and research beneficiary audiences; it could be used to underpin co-production research reporting guidelines or to contribute to teaching good practice to students or colleagues new to co-production.

**Fig. 3:**
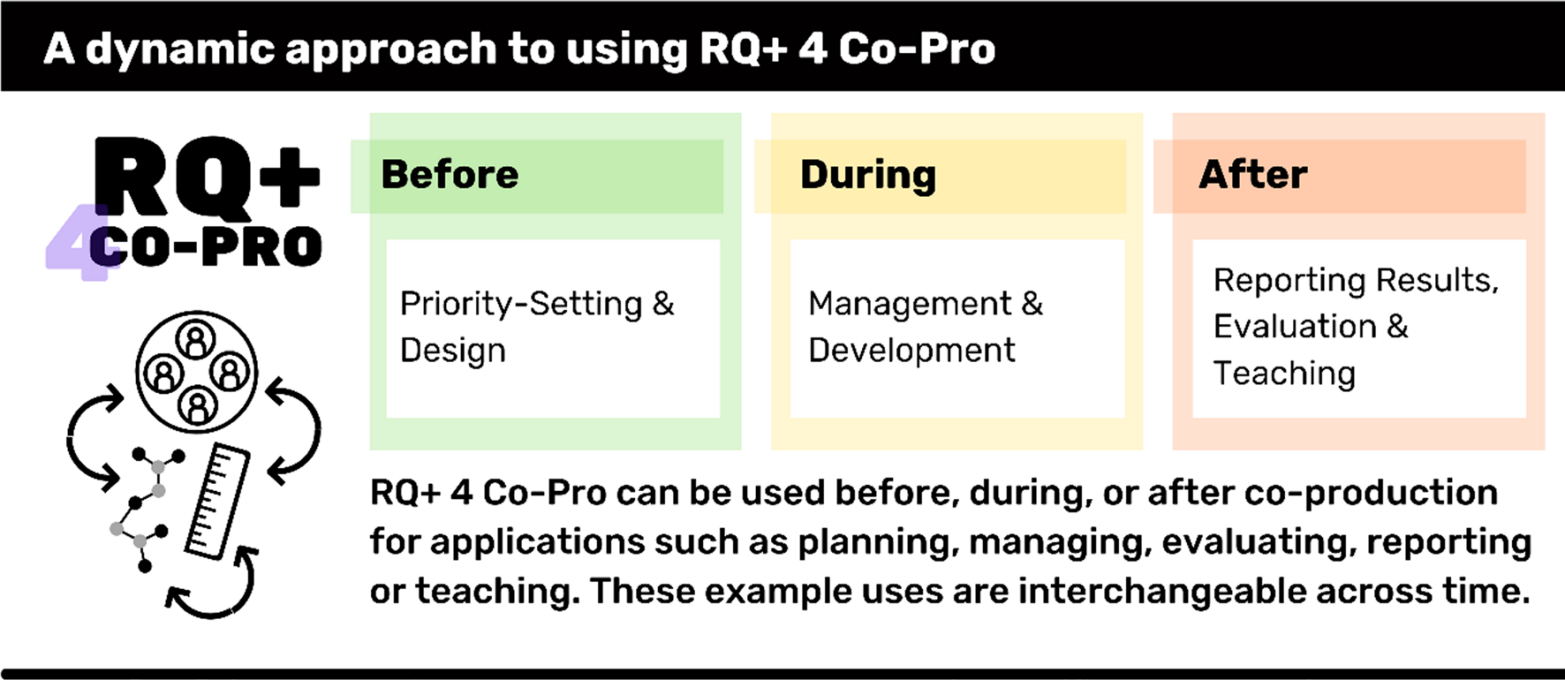
Presents illustrative examples of RQ + uses across the co-production lifecycle

All these uses of RQ + 4 Co-Pro before, during, and after a research project, suggest the need for specialized tools and language suitable to the relevant audience. Some beneficiaries of co-production—such as patients or community groups—may require significant tailoring of the Assessment Instrument prior to use. We also note the importance of tempering interpretations of results of RQ + 4 Co-Pro evaluations by considering whether they are external evaluations or self assessments. Both may be valid and useful applications, but incentives should be considered alongside results. Furthermore, we highlight results of this study were reached with a sample of expert co-production specialists. This may have affected the results and diversifying co-production experience should be considered in new applications or further testing (Fig. 3).

### New applications can, and should, drive RQ + 4 Co-Pro adaptation and improvement

Our study results provide a first version and endorsement of the RQ + 4 Co-Pro Framework and Assessment Instrument. However, we encourage flexibility and adaptation in future uses. The specific contextual factors and quality dimensions represented in this publication offer a robust starting point, but new users should be mindful of their own values and objectives and ensure these are embodied in the evaluation framework they put to use. This recommendation is aligned with recent literature which makes the case for refining models and frameworks through subsequent research and application rather than treating them as reified and unchangeable products [33], particularly when supporting patient and public involvement in research [8].

This study confirms the experience of IDRC and their experience with the RQ + approach. The critical elements to replicate are three tenets: (1) context matters, (2) quality is multi-dimensional, and (3) systematic and empirical appraisal. How these three tenets are operationalised is a context-dependent decision. One particularly interesting implication is how funders or publishers might deal with the strong support for the value of the dyadic qualitative interview in the field-test (a representation of the third tenet). Indeed, the elaboration of contextual detail and reasoning for decision-making was reported by our study participants as enriching, and in cases essential, to the quality and accuracy of their assessments. It is possible funders and publishers could see similar benefits by including primary data in their assessment procedures. How to build the required resources and ethical parameters for engaged assessment into the work of funders and publishers is territory requiring further exploration.

Overall, we suggest adaptations to the Framework and Assessment Instrument in new settings and contexts will contribute to RQ + 4 Co-Pro improvements. We believe adaptations will lead to improvements for co-production research, as no single evaluation framework will represent co-production quality in perpetuity. Co-production is a dynamic exercise, and so too must be its evaluation.

### Study limitations

We note four main limitations of our study. Limitations relate to the field-test implementation and to the transferability of findings. *First,* we’ve suggested RQ + 4 Co-Pro will be useful to research co-production broadly. That is to say, we hold the view co-production is an umbrella term that can be inclusive of the many idiosyncratic approaches to research undertaken with those who will use or benefit from it. However, our study sample was limited to participants and projects from the Integrated Knowledge Translation Network, all of whom are primarily health researchers. This may limit transferability to co-production under alternative nomenclature and into other disciplines. This was counterbalanced by engaging an international team of researchers working in different co-production traditions. No study participant we interviewed suggested transferability was limited.

*Second,* given all participants in the study are experienced co-production researchers and members of the same health research network (IKTRN), we note that working within an established group may present social desirability bias. This bias may have manifested in the dyad assessments in the field-test, or the follow-up interviews regarding the relevance and utility of RQ + 4 Co-Pro. We acknowledge this as a potential limitation as readers interpret the results. That being said, we have limited concern that social bias has affected the field-test, as we did not collect or utilize dyad evaluation scores. Indeed, the purpose of this study was to assess the relevance and utility of the RQ + 4 Co-Pro Framework, not to draw a final conclusion about the quality of the projects or researchers sampled. Participants may have felt the need to defend their own or their colleagues’ projects, but participants held no direct stake in the outcome of the RQ + 4 Co-Pro field-test.

*Third*, our field-test only engaged principal investigators (i.e., researchers) within the dyadic evaluations, which may have limited what we learned through interviews. These participants volunteered and their projects were self-selected which could lead to some bias towards higher quality projects. We encourage follow-up work on RQ + 4 Co-Pro to focus on refining the Framework and Assessment Instrument with research beneficiaries specifically. We also encourage co-production beneficiaries who were not included in our sampled participants or co-production team to be engaged (for example, patients, relatives, community activists, amongst others).

*Fourth*, our sample contained at least two demographic occurrences/biases. On one hand, all participants in the study, and members of our co-production team, are currently based in high-income countries. On the other, all study participants self-identified as women. These are significant considerations for readers to understand as they interpret results. Further tests of RQ + 4 Co-Pro with a more diverse participant group (inter alia, gender and geography) would serve to strengthen confidence in the Framework and Assessment Instrument’s relevance and transferability.

## Conclusion

This paper presents a co-produced and field-tested framework for the evaluation of research co-production. Our study shows RQ + 4 Co-Pro can be both relevant and useful for the evaluation of co-production, and we are confident the diversity of expert perspectives engaged in its development positions the RQ + 4 Co-Pro Framework well for future uptake and use. We encourage co-production stewards of all-types—researchers, funders, universities, journals, to name a few—to experiment with their own applications of RQ + 4 Co-Pro. We encourage those who do to adapt RQ + 4 Co-Pro to their specific purpose, and to share their experience doing so.

This study has reinforced the importance of the three RQ + tenets for co-production evaluation. *First*, context is an inseparable component of any co-production endeavour. We will learn more from accepting and studying the context where co-production occurs than we will from blinding and isolation. *Second*, co-production quality is a multi-dimensional concept that requires a similarly holistic approach to evaluation. At the same time, our study indicates balancing assessments of rigour, legitimacy, and positioning for use is both possible and essential. *Third*, co-production evaluation should rely on the same standards for evidence as co-production research itself. This means moving evaluations beyond the opinion of a peer (almost always a scientist, not a research beneficiary), and requiring empirical evidence collection and systematic and transparent evaluation. In the future, data collected and stored on co-production contexts and quality dimensions will serve rigorous scientific study of the barriers and enablers of co-production’s societal impacts.

We know more critical and rigorous evaluation is necessary for understanding and improving co-production, and, for ensuring co-production delivers on its promise of better health, health equity, and societal good. RQ + 4 Co-Pro is one immediately practical step in this direction.

## Supplementary Information

**Additional file 1. **RQ+ 4 Co-Pro Assessment Instrument.

**Additional file 2. **SRQR checklist_RQ+ 4 Co-Pro.

## Data Availability

Please contact the corresponding author.

## Abbreviations

RQ+: Research Quality Plus
RQ + 4 Co-Pro: Research Quality Plus for Co-Production
CIHR: Canadian Institutes of Health Research
IDRC: International Development Research Centre
GCRF: Global Challenge Research Fund
I: Interviewee
IKT: Integrated knowledge translation
IKTRN: Integrated Knowledge Translation Research Network
