# Supplementary material for: Evaluating the quality of research co-production: Research Quality Plus for Co-Production (RQ + 4 Co-Pro)
Source: Health Res Policy Syst. 2023 Jun 13;21:51. doi: 10.1186/s12961-023-00990-y (PMC10262138; doi:10.1186/s12961-023-00990-y)
Supplement: Supplementary file 1 — Additional file 1. RQ+ 4 Co-Pro Assessment Instrument. [file 12961_2023_990_MOESM1_ESM.pdf]

RQ+ 4 CO-PRO

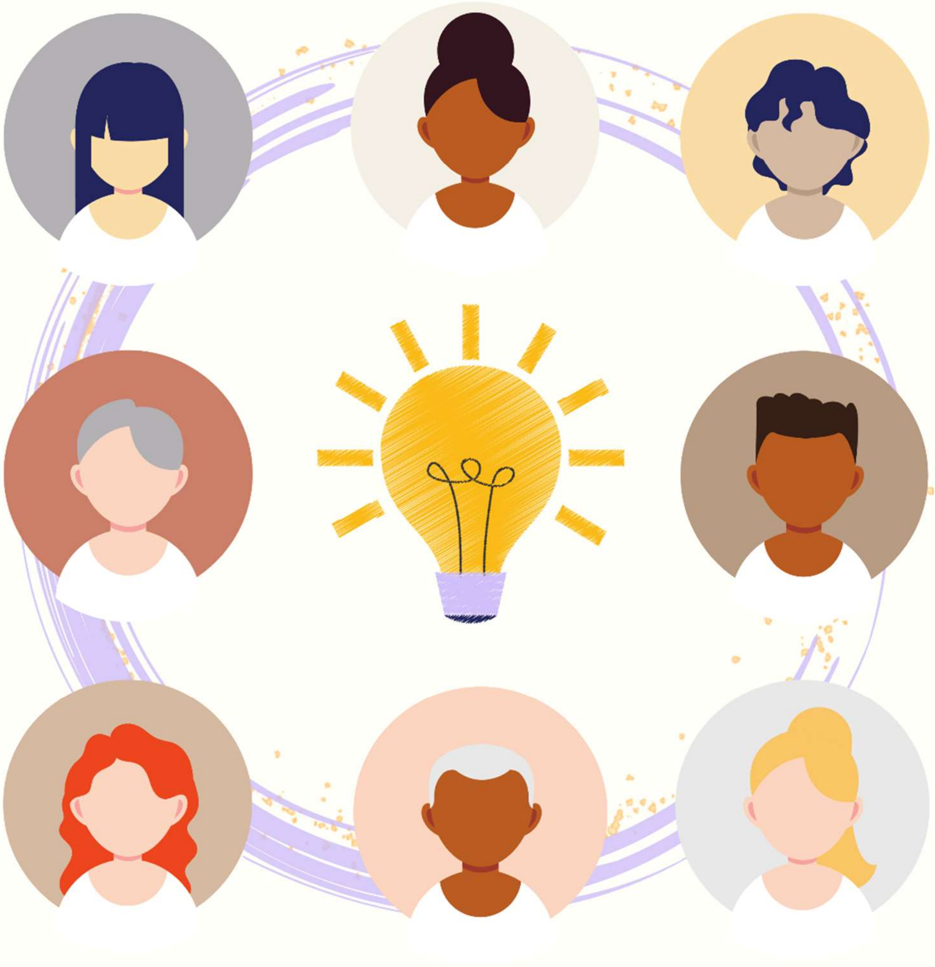

Research Quality Plus *for* Co-Production

Assessment Instrument

A practical and holistic tool for evaluating the quality of research co-production

Authors

Robert McLean
International Development Research Centre
& Ottawa Hospital Research Institute

Fred Carden
Using Evidence Inc.

Alice Aiken
Vice President Research & Innovation,
Dalhousie University &
Canadian Institutes of Health Research

Rebecca Armstrong
National Disability Insurance Agency,
Australia

Judy Bray
Vice President Research,
Canadian Cancer Society

Christine Cassidy
Dalhousie University

Olivia Daub
Western University

Erica Di Ruggiero
University of Toronto

Leslie Fierro
McGill University

Michelle Gagnon
Independent Consultant

Alison Hutchinson
Deakin University

Roman Kislov
Manchester Metropolitan University
& University of Manchester

Anita Kothari
Western University

Sara Kreindler
University of Manitoba

Chris McCutcheon
Ottawa Hospital Research Institute

Jessica Reszel
Ottawa Hospital Research Institute
& University of Ottawa

Gayle Scarrow
Michael Smith Health Research
British Columbia

Ian Graham
University of Ottawa &
Ottawa Hospital Research Institute

Suggested Citation: McLean R, Carden F, Aiken A, Bray J, Cassidy C, Daub O, Di Ruggiero E, Fierro L, Gagnon M, Hutchinson A, Kislov R, Kothari A, Kreindler S, McCutcheon C, Reszel J, Scarrow G, Graham I. (2023) *The Research Quality Plus for Co-Production (RQ+ 4 Co-Pro) Assessment Instrument.* Integrated Knowledge Translation Research Network. Ottawa, Canada.

IKT
Research Network

Assessment Instrument

"**Research Quality Plus for Co-Production** (RQ+ 4 Co-Pro) is an approach to assessing the quality of research co-production. RQ+ 4 Co-Pro prioritizes the importance of a meaningful researcher – knowledge user partnership throughout research design, research conduct, and sharing of research findings. The aim is to advance rigorous, legitimate, and useful knowledge co-production." (McLean, Graham & Carden 2022)

This document presents the RQ+ 4 Co-Pro Assessment Instrument. The Assessment Instrument provides guidance, detailed descriptors, record-keeping tables, and includes rubrics that will facilitate the use of the RQ+ 4 Co-Pro Framework in a research co-production evaluation. It is an open access tool that users are encouraged to adapt to their purpose and context.

Additional resources:

A research report in *Health Research Policy and Systems* outlines the results of the RQ+ 4 Co-Pro field-test, and discusses potential uses and users of RQ+ 4 Co-Pro.

A Study Protocol in *Implementation Science Communications* describes the design of the field-test used to first apply, evaluate, and develop the RQ+ 4 Co-Pro Framework and Assessment Instrument. In open access here.

A concept chapter in the book *Research Coproduction in Healthcare* positions RQ+ 4 Co-Pro in the evaluation and co-production literature, and outlines the genesis of the Framework and Assessment Instrument. Read it here.

The International Development Research Centre's dedicated Research Quality Plus website collates open access resources and publications on the RQ+ approach. Visit in English, French or Spanish.

What is RQ+ 4 Co-Pro?

RQ+ 4 Co-Pro is built on the three tenets of the RQ+ approach designed and implemented by the International Development Research Centre and its research and evaluation community [www.idrc.ca/RQplus]. The RQ+ tenets are:

1- Context matters. Embrace context as central to understanding the quality of any research.
2- Quality is a multi-dimensional construct, and it should relate to the values and objectives underpinning the work.
3- Judgements of research quality should be empirical and systematic. Not only peer-opinion.

RQ+ 4 Co-Pro embraces these tenets and applies them to the specifics of the field of research co-production. The result is the novel RQ+ 4 Co-Pro Framework. The figure on the following page presents the RQ+ 4 Co-Pro Framework in a 1-page infographic, outlining its key components: 1) contextual factors, 2) quality dimensions and sub-dimensions, and 3) data collection and appraisal processes.

The concepts and definitions provided here were first developed in a concept chapter entitled *Evaluating Research Coproduction* in the book *Research Coproduction in Healthcare* (see: McLean, Graham & Carden, 2022).

Following this prototype publication, the RQ+ 4 Co-Pro Framework and Assessment Instrument were revised based on international field-testing carried out using 18 discrete research co-production projects and overseen by a team of co-production scientists and stewards. The protocol for this testing and development is available in open access for users of this instrument who are interested in the RQ+ 4 Co-Pro Framework's conceptual grounding and practical development (McLean et al. 2022).

RQ+ 4 CO-PRO

Research Quality Plus for Co-Production

RQ+ 4 Co-Pro is an approach to defining and evaluating the quality of co-production. It allows tailoring to context, values, and purpose. It can support planning, management and learning across the lifecycle of a co-production project, program or organization.

The RQ+ 4 Co-Pro Framework

1. Contextual Factors

1. Knowledge Use Environment

2. Research Environment

3. Capacities for Co-Production

2. Quality Dimensions & Sub-Dimensions

1. Rigour
- 1.1 Design
- 1.2 Methodological Integrity

2. Legitimacy
- 2.1 Inclusion of Local Knowledge & Ways of Knowing
- 2.2 Trust, Power & Mutually Beneficial Partnerships
- 2.3 Intersectionality
- 2.4 Attention to Potential Negative Consequences

3. Positioning for Use
- 3.1 Relevance
- 3.2 Openness & Actionability

3. Empirical & Systematic Appraisal

Characterization of each contextual factor, dimension and sub-dimension is done using tailored rubrics. These rubrics combine quantitative and qualitative measures and draw on empirical evidence.

Quality ratings are made on an 8-point scale showing four levels of performance (or progress), and contextual factors are placed on a 4-point rubric indicating a categorical assessment. This is just one example. Scales should be designed to fit a purpose.

A Dynamic Approach

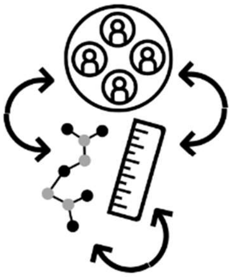

Before

Priority-Setting & Design

During

Management & Development

After

Reporting Results, Evaluation & Teaching

The RQ+ 4 Co-Pro Assessment Instrument, and how to use it.

This Assessment Instrument provides guidance, record-keeping tables, and includes rubrics that will facilitate the use of the RQ+ 4 Co-Pro Framework in a practical evaluation exercise.

Each contextual factor and quality dimension is subject to evaluation by collecting and interpreting primary and secondary data from a variety of sources. This data is appraised using common rubrics. Rubrics for contextual factors are set as categories, and higher or lower ratings do not imply better or worse. These rubrics are a means of categorizing the enabling or constraining environment. Rubrics for quality dimensions do facilitate an evaluative judgement by describing what different levels of quality should look like, with ratings ranging from unacceptable to very good. Sub-dimensions can be weighted to determine a final or overall score in any assessment, when helpful and appropriate to do so.

In the pages that follow, there are definitions of these elements (contextual factors, dimensions and sub-dimensions), as well as indication of the meaning of scoring values. It would be impossible to cover every eventuality as each co-production effort and context is unique. The definitions provided are therefore broad. On the ratings sheet, note that a space for comment is provided at each juncture. Recording this short qualitative justification for your quantitative rubric rating is an important part of the assessment process, and the data may be just as useful as any numerical ratings. Other uses of RQ+ 4 Co-Pro, such as for research design, monitoring, or teaching may require additional adjustments to both the Framework and Assessment Instrument. The language used here describes a completed co-production.

Tips for using the Assessment Instrument:

1. Because co-production research is varied and carried out in partnership with a range of different communities, the definitions presented here may need context-driven adaptation prior to implementation. Speak to stakeholders about your application and fit the Assessment Instrument to purpose.

2. Critical to successful assessment is clarity and specificity of the evaluand. The evaluand may be a single research project, a program of research, the body of research supported by an organization, it may also be an assessment of the body of research of an individual researcher. Be transparent and purposeful about the object of evaluation, and efficiency and rigour of the Assessment Instrument will be greatly improved.

3. Plan your data collection process. The partnership aspect of co-production means that a conversation with the relevant stakeholders – research beneficiaries and researchers – is an important element of the assessment. As well, it is necessary to use judgement, as with any rubric, several factors are considered in a rating. For that reason, the justification section for each rating is important to include.

RQ+ 4 Co-Pro Assessment Instrument

Standard Data

Date of evaluation:

Subject of evaluation
(project, program, organization, individual):

Location(s):

PI or Co-PIs
Name:
Organization:
Contact information:

Knowledge Users:
Name:
Organization:
Contact Information:

Evaluator(s):
Names:
Contact information:

Evidence-base

1. Documents consulted:
2. People consulted (and roles as researcher, KU, donor, etc.):

Part 1 – Categorizing the Context

Contextual Factors

Co-production research always occurs in political, economic, social, and scientific settings. RQ+ 4 Co-Pro identifies three contextual factors that can be monitored and categorized in a co-production project or program evaluation. By studying these factors, users of RQ+ 4 Co-Pro can learn, share experience, and cultivate enabling environments for co-production work. In the more immediate term, categorizing research context can help project coordinators, funders, or managers understand risk factors and identify mitigation strategies for individual projects or for monitoring project portfolios. Classifications of context are done independently of ratings against the quality dimensions, and they are not intended to modify project quality ratings. The intent is to document and understand the project environment. One categorization is not meant to imply 'better than' another categorization. The three RQ+ 4 Co-Pro contextual factors are: 1) Knowledge Use Environment; 2) Research Environment; 3) Capacities for Co-Production. Each is defined and a rubric provided.

1. Knowledge Use Environment

This contextual factor addresses the absorptive capacity of the knowledge use environment. This typically stems from the broad environment and culture of the knowledge-user partner and then manifests for the co-production team. The knowledge use environment may be highly empowering, with a strong appetite for research, evidence, or knowledge, to inform policy, program, practice or product improvement. Here, resources and incentives will encourage and reward the use of evidence in decision-making. Alternatively, the environment may be restrictive; as a result, the co-production team faces significant barriers, even professional risks, to research evidence vis-à-vis alternative decision-making approaches. In a restrictive environment, resources and incentives do not support research uptake and use.

1 (Restrictive)	2 (Unsupportive)	3 (Supportive)	4 (Empowering)
There is disinterest in or opposition to the use of research. Research, evidence, or knowledge processes and/or products are overlooked or challenged.	While there may be a claim of the importance of using evidence, there is limited evidence that it is valued in decision-making processes. There may be a lack of awareness as to the role or utility of research, evidence or knowledge.	There is support for the importance of using research, evidence, or knowledge, and an awareness of how it might improve decision-making. Research, evidence or knowledge is generally valued and does not face avoidance and/or skepticism.	There is clear demand for research, evidence or knowledge. There is active encouragement for research, a thorough understanding of its requirements, and a demonstrable intent to use evidence to inform decisions and directions.
Rating:			
Comments & Justification			

2. Research Environment

This contextual factor addresses the environment in which the researcher partner(s) in the co-production team works. In some circumstances the environment may empower co-production as a valid means of knowledge generation and provide researchers incentives, resources, and rewards for good practice. Alternatively, co-production may be an undervalued or diminished means of conducting research or generating knowledge, where researchers may be explicitly or implicitly discouraged from undertaking co-production and thus put career progression and peer acceptance at risk by engaging knowledge users in their research.

1 (Restrictive)	2 (Unsupportive)	3 (Supportive)	4 (Empowering)
There is disinterest or opposition to co-production research. Co-production processes and/or products are dismissed for low credibility, and this may negatively influence perceptions of those undertaking co-production vis-a-vis their peers. This may be explicit or implicit.	While there are no explicit negative assumptions about co-production, there is limited evidence that co-production is valued similarly to traditional curiosity-driven science. There may be instances of implicit bias against co-production work, and a lack of awareness as to the specificities of co-production from peers and institutional leadership.	There is support in the research environment for co-production work. Co-production results are valued similarly to curiosity-driven science. The process is encouraged and supported by peers. Co-producers do not face career or status setbacks.	Co-production research is incentivized, resourced and valued. Co-producers' results and processes are valued equally to other forms of science, and when done well, encouraged as a legitimate and rigorous means of knowledge generation. Co-producers receive incentives and rewards for good work.
Rating:			
Comments & Justification:			

3. Capacities for Co-Production

This contextual factor categorizes the extent to which the research places focus on training and developing co-production practice and/or theory amongst researchers and knowledge users. Capacity/capability (we use the terms interchangeably) strengthening effort is tracked because co-production is a young and emerging field, and nurturing the next generation is required for future acceptance and sustainability. When the focus is strong, a considerable amount of time and resources are devoted to purposefully and consciously developing the skills of team members and aptitude for co-production is envisaged as a positive result of the effort (amongst both researchers and knowledge users). Alternatively, capacity building in co-production may not be an explicit part of the research effort but may be implicit through the engagement of junior or new co-production researchers (e.g., PhD candidates) or knowledge users new to co-production research. A lesser focus is identified when no discernable resources are devoted to strengthening capabilities/capacities for co-production, and the only viable skill development opportunity for researchers or knowledge users will come from learning by doing. This may be the case with a highly experienced or beginner co-production team. Unlike the other contextual factors, Capacities for Co-Production does not denote a measure of risk. This is not an outcome measure in this instrument Rather, It is a measure of the intensity of the effort.

1 (No Focus)	2 (Minimal Focus)	3 (Significant Focus)	4 (Strong Focus)
The team has no access to support (training, mentors, resources, etc.) to develop co-production skills. No documentation indicates co-production capacity strengthening is a goal or objective of the work. It is possible capacity is/was built, but this was clearly an unintentional outcome of the project.	The team may receive general capacity building, but little if any, addresses co-production specific skills. Through 'learning by doing' some capacity strengthening is likely to occur.	There is evidence to indicate a capacity strengthening plan was developed for/by the project/program, and it has been enacted. The team is learning by doing and through training. There are demonstrable capacity strengthening outcomes expected.	There is a clear co-production learning plan built into the project/program, and meaningfully enacted. The team places strong focus on strengthening co-production capacities of the knowledge users and researchers specifically through the process, and capacity strengthening is envisioned as an important objective and outcome of the project (regardless of this outcome being achieved yet.)
Rating:			
Comments & Justification:			

Part 2 – Assessing Co-Production Qualities

Quality Dimensions and Sub-Dimensions

Any judgement of research quality should reflect the values underpinning that research effort. RQ+ 4 Co-Pro articulates three dimensions, and eight corresponding sub-dimensions of quality that reflect broad values for partnered, stakeholder-engaged research.

Co-production research must be robust and methodologically sound, thus we begin with ‘Rigour’ – a non-negotiable component of any co-production effort. The second dimension of RQ+ 4 Co-Pro, Legitimacy, highlights four sub-dimensions that together measure the fidelity of the co-production effort to the environment in which it occurs and the results it will produce for intended beneficiaries. The third dimension, Positioning for Use, examines the relevance of the research to the needs of users and the openness and actionability of the process and results.

In the RQ+ 4 Co-Pro Framework these dimensions are not independent variables they are interrelated. Yet, by disaggregating and allowing focus on each component, RQ+ 4 Co-Pro highlights and brings importance to the alternative and diverse qualities that ultimately underpin excellence in co-production. For our purposes the dimensions hold equal weight. Other users may choose to weigh dimensions differently in order to increase focus on challenging or significant components of their work. Working from lowest rating to highest, the eight-point rubric, is as follows:

Insufficient information to assess	Unacceptable		Less than acceptable		Good		Very good	
IIA	1	2	3	4	5	6	7	8

1. Rigour

The first dimension of research co-production quality addresses the technical merit and demonstrable excellence of the work according to the approach to knowledge generation appropriate to the partners involved. This requires an examination of the project vis-à-vis the standards and expectations of the methodological approach (qualitative research, clinical trials, statistical methods, ethnographic immersion, for example). Meaningful co-production partnerships are considered across any and all fields and knowledge generation traditions. This part of the assessment must be considered vis-à-vis the intentions of the work and the fair expectations of the knowledge-user partners. In some circumstances engagement will be necessary from start to finish, in other cases knowledge users and researchers will have negotiated mutually beneficial terms, and these idiosyncrasies should be considered and examined here. The dimension is represented with two distinct sub-dimensions.

1.1. Design

This measure of quality addresses the design of the effort using the accepted best practices of the field. This examines how the study is framed in the current knowledge, reproducibility of the design, how methodological standards are met or exceeded with viable innovations, and the overall design openness. This dimension also considers the co-production process, including when and how knowledge-user and researcher engagement is planned, and whether adaptation or emergence are built in.

<table>
<tr><td>Insufficient information to assess</td><td colspan="2">Unacceptable</td><td colspan="2">Less than acceptable</td><td colspan="2">Good</td><td colspan="2">Very Good</td></tr>
<tr><td rowspan="2">IIA</td><td>1</td><td>2</td><td>3</td><td>4</td><td>5</td><td>6</td><td>7</td><td>8</td></tr>
<tr><td colspan="2">The design is not acceptable to meet the standard of its field of work. It may represent a wasteful (duplicative, unusable etc.) study.</td><td colspan="2">The design should have been revised to address important gaps or weaknesses</td><td colspan="2">The design is acceptable. It is well grounded in the current knowledge base, positions the work to produce relevant and useful knowledge.</td><td colspan="2">The design is clear, thorough and transparent. It represents a solid and well thought-out design in its domain.</td></tr>
<tr><td colspan="9">Rating:</td></tr>
<tr><td colspan="9">Comments & Justification:</td></tr>
</table>

1.2 Methodological Integrity

Methodological integrity refers to the technical fidelity of research implementation and research management decisions. As understood in co-production research, this embraces necessary and agreed adaptations and emergent designs. This will include how principles of working practice (ie. partnering) are established and navigated by the full co-production team. How partnerships are managed is essential throughout each part of the process, which will typically examine issues such as: (i) research questions are pursued rigorously (ii) adequate and appropriate data collection is conducted, (iii) relevant analysis frameworks are selected and applied according to best practice and knowledge-user needs, (iv) conclusions are grounded in data collected, and, (v) clear and accurate presentation of results in light of knowledge-user contexts and needs.

<table>
<tr><td>Insufficient Information to Assess</td><td colspan="2">Unacceptable</td><td colspan="2">Less than acceptable</td><td colspan="2">Good</td><td colspan="2">Very Good</td></tr>
<tr><td rowspan="2">IIA</td><td>1</td><td>2</td><td>3</td><td>4</td><td>5</td><td>6</td><td>7</td><td>8</td></tr>
<tr><td colspan="2">The design was not followed, and no discernable or reasonable justification was provided. Negotiated partnering terms/principles were entirely absent or coerced by one group on another.</td><td colspan="2">Significant changes to the research were made outside the design with limited rationale. Negotiated partnering principles were weak or established but ignored.</td><td colspan="2">The conduct of the study generally followed the design and most adaptations were reasonable and could be justified. Negotiated partnering principles were evident and largely followed.</td><td colspan="2">The conduct of the study followed the design well. It clearly rationalized any adaptations required by new knowledge of, or changes in, context or emerging issues in the coproduction process. Negotiated partnering principles were clear, owned, and enacted by all partners.</td></tr>
<tr><td colspan="9">Rating:</td></tr>
<tr><td colspan="9">Comments & Justification:</td></tr>
</table>

2. Legitimacy

Legitimacy addresses the fidelity of the co-production to the context in which it is or will be implemented. In the context of co-production, legitimacy includes sub-dimensions related to fairness and meaning in knowledge generation, diversity, equity and inclusion, and meaningful relationships being created and/or sustained between all partners involved in the co-production effort. Specifically, Legitimacy is represented in four sub-dimensions.

2.1 Inclusion of Local Knowledge and Ways of Knowing

This sub-dimension addresses the degree to which the research is grounded in the reality and knowledge base of the intended users and beneficiaries of the work. Exemplary projects will ensure scientific methods embrace and empower the realities of local ways of knowing, existing cultures, and norms or expectations about knowledge. These could be cultural, commercial, organizational, or political knowledge localities, depending on the aims and context of the project. Attention must be paid to decolonizing local standards from predominant scientific standards surrounding knowledge and evidence, and appropriately weighing all partners' perspectives.

<table>
<tr><td>Insufficient Information to Assess</td><td colspan="2">Unacceptable</td><td colspan="2">Less than acceptable</td><td colspan="2">Good</td><td colspan="2">Very Good</td></tr>
<tr><td rowspan="2">IIA</td><td>1</td><td>2</td><td>3</td><td>4</td><td>5</td><td>6</td><td>7</td><td>8</td></tr>
<tr><td colspan="2">The research ignores local realities and knowledge. It implements a methodology driven by external interests and experience.</td><td colspan="2">The research is insufficiently grounded in local realities and knowledge and significant gaps remain.</td><td colspan="2">The research is sufficiently grounded in local realities and integrates local knowledge with some inconsistencies.</td><td colspan="2">The research is fully grounded in local realities and context, integrating relevant local knowledge and embraces ways of knowing relevant to the users and beneficiaries.</td></tr>
<tr><td colspan="9">Rating:</td></tr>
<tr><td colspan="9">Comments & Justification:</td></tr>
</table>

2.2 Trust, Power, and Mutually Beneficial Partnership

This sub-dimension examines the underlying power dynamics of the research process, specifically examining how power was created, shared, and sustained. It also interrogates if/how the co-production effort is designed and managed to address the needs and desires of all parties throughout the research process. A mutually beneficial partnership does not mean all tasks and resources are shared equally; it means decisions about how tasks and resources are utilized are mutually decided.

<table>
<tr><td>Insufficient Information to Assess</td><td colspan="2">Unacceptable</td><td colspan="2">Less than acceptable</td><td colspan="2">Good</td><td colspan="2">Very Good</td></tr>
<tr><td rowspan="2">IIA</td><td>1</td><td>2</td><td>3</td><td>4</td><td>5</td><td>6</td><td>7</td><td>8</td></tr>
<tr><td colspan="2">The power imbalances in the research and the research team are not addressed.</td><td colspan="2">There is some evidence that efforts were made to negotiate the partnership, but significant problems were encountered in implementation, suggesting inadequate negotiation.</td><td colspan="2">There is clear evidence the partnership was negotiated and freely agreed by both researchers and knowledge users and was largely sustained in the research process with some challenges.</td><td colspan="2">There is clear evidence that the partnership was negotiated and freely agreed by both researchers and knowledge users. It has been sustained throughout the research process.</td></tr>
<tr><td colspan="9">Rating:</td></tr>
<tr><td colspan="9">Comments & Justification:</td></tr>
</table>

2.3. Intersectionality

Intersectionality considers the wide range of social identities that construct our lived experience and the power differentials related to these identities and locations. Accordingly, this sub-dimension addresses the degree to which the work takes account of the varied perspectives, and produces equitable processes and outcomes for different intersectional connections. Issues of diversity, equity, and inclusion are considered here. Intersectionality is a critical element in each of the design and implementation components of the work and the assessment should focus on the extent to which intersectionality is considered and built into each phase of the project. In the case of an impact assessment, it may examine outcomes for varied intersectional groups. In the case of a needs assessment, it may examine whose needs are being considered and whose are not, or how they are being valued and why. For co-production, intersectionality should be considered in the positionality and interactions of the team undertaking the project, and in the implications and effects the effort has on participants and society more broadly. Very good co-production will be sensitive to the social environment in which the research takes place and cognizant of the potential biases the co-production team brings into the work. No co-production project should be blind to power and influence considerations.

Insufficient Information to Assess	Unacceptable		Less than acceptable		Good		Very Good	
IIA	1	2	3	4	5	6	7	8
	The co-production does not address intersectionality sufficiently to be acceptable. Generally, it is blind to issues of gender, culture, class, and other intersections which should be pertinent to the meaning of the work. Weakness applies to both the team approach and the research.		Although some attempts were made, consideration of intersectionality is poor or underdeveloped. Neither the co-production team or the co-production effort demonstrate sufficient attention to power interactions and their implications.		The co-production identifies which intersectionalities are addressed and takes adequate account of most relevant intersectionality issues in team dynamics but also in research analysis and reporting.		The team dynamics and the research approach take account of relevant intersectionality considerations and are responsive to new considerations that may have emerged. The research goes well beyond disaggregation of data in analysis or reporting, and makes intersectional considerations a component of the choice of questions, methods, analytical theories and frameworks and reporting/sharing approaches.	
Rating:								
Comments & Justification:								

2.4. Attention to Potential Negative Consequences

Although the benefits of co-production are widely proclaimed, the approach can yield negative consequences on those who do it and those it intends to benefit. This sub-dimension refers to the strategies employed in the co-production project to anticipate, track, minimize and mitigate any negative consequences of the work. Negative consequences could include damages to individual partners or their organizations due to their participation in the partnership, damages to participants, adverse outcomes for beneficiary individuals or communities, or damages to the natural environment. Evidence of exemplary performance is found in an ethical approach throughout the research co-production process, but also in the way user/beneficiary relationships are managed and how these perspectives are valued in how decisions about project progress are made.

Insufficient Information to Assess	Unacceptable		Less than acceptable		Good		Very Good	
IIA	1	2	3	4	5	6	7	8
	The design does not anticipate or track for potential negative consequences on team members, participants, or intended beneficiaries.		The partnership has poor systems for anticipating and tracking for potential negative consequences on team members, participants, or intended beneficiaries.		The partnership adheres to an externally reviewed ethics approach and has included measures to anticipate, monitor and mitigate negative consequences that may affect team members, participants, or intended beneficiaries.		The partnership utilizes an externally approved and exemplary ethical approach and is vigilant about and responsive in identifying potential negative consequences for team members, participants, as well as all who may have been impacted by the work – including those it intended to benefit and any unintended but impacted people, communities, or environments.	
Rating:								
Comments & Justification:								

3.Positioning for Use

Positioning for Use means that the research is designed, carried out, and shared in ways which promote its use by the communities it is intended to benefit. It addresses the extent to which the co-production process enhanced the likelihood of research uptake and impact. A first critical element is how relevant the research objectives and questions are for the intended beneficiaries and/or users of the work. Second is the creation of audience-friendly and open access research outputs and results. User engagement as a means of facilitating knowledge translation is a matter of scientific rigour in co-production: thus, it is assessed specifically under Quality Dimension one, Rigour.

3.1. Relevance

Relevance reflects the extent to which the research takes on existing and predominant societal or practical problems of relevance to knowledge users and beneficiaries. The measure examines how the research was prioritized, who framed it, who it serves, and how widely endorsed the needs and challenges it addresses are by co-producers and the organizations or communities that are or will be affected/served by the outcomes.

Insufficient Information to Assess	Unacceptable		Less than acceptable		Good		Very Good	
IIA	1	2	3	4	5	6	7	8
	The research has no relevance to or engagement with the local community/user/ intended beneficiary.		The topic and approach is only marginally relevant and should be revisited with beneficiaries and knowledge users.		The research topic is relevant to knowledge users and serves a community need. (However, maybe not the highest need for beneficiaries in the affected community.)		The research is highly relevant to knowledge users and beneficiaries, and is clearly intended to serve their and their communities' needs.	
Rating:								
Comments & Justification:								

3.2. Openness & Actionability

This sub-dimension addresses how research is conducted and how results are tailored into outputs and results that are useful, attractive, and understandable for knowledge users and beneficiaries. The useability of the solution generated is considered, and so is the presentation of the solution in an engaging format. This includes how openly available (open access), applicable, tailored, and timely the conduct and results are for action.

Insufficient Information to Assess	Unacceptable		Less than acceptable		Good		Very Good	
IIA	1	2	3	4	5	6	7	8
	The presentation of findings does not address issues of accessibility for intended audiences, and the needs of users.		The presentation of the findings is limited and not meaningful for all intended users. The majority of sharing efforts may have privileged one group (e.g., academic priorities).		The findings are available, clear and presented in formats and language that are usable by all intended audiences.		The findings are available, clear, and presented in formats and language that are usable by all intended audiences. Partners have worked together to identify relevant users and audiences, and have co-created a sharing plan including tailored sharing strategies for identified groups as appropriate.	
Rating								
Comments & Justification:								

Potential uses & users of RQ+ 4 Co-Pro

RQ+ 4 CO-PRO

Applying RQ+ 4 Co-Pro

RQ+ 4 Co-Pro can support the design, management and evaluation of research co-production. It can be used at any stage of the co-production life-cyle.

When	Use	Motive
Before	Establishing expectations, priorities and meaningful partnerships<br>Developing principles and shared values<br>Co-production project design<br>Grant application review	RQ+ 4 Co-Pro can provide an agenda for fair and transparent terms of engagement. The contextual factors and quality dimensions can and should be adapted to the needs of the effort at hand. When they are set, they identify/define clear priorities and parameters for what is considered good in the work. This priority-setting exercise can be used to communicate objectives and values of importance to the user.
During	Monitoring and managing progress<br>Formative or developmental evaluation<br>Sharing and reporting protocols and processes	RQ+ 4 Co-Pro presents an approach for regularly revisiting shared values and objectives as well as course correcting when needed. Whether RQ+ 4 Co-Pro is applied as a self-assessment checklist, as a formative evaluation framework, or in any mid-term format, the approach can provide a clear learning agenda and systematic means of risk management for the user.
After	Publication review<br>Summative evaluation of a project or portfolio of projects<br>Supporting impact assessment<br>Teaching, capacity strengthening and field-building	RQ+ 4 Co-Pro provides a retrospective assessment approach that can shine new light on the benefits, limitations, and areas for improvement in/of research co-production. By selecting evaluative criteria that reflect the values co-producers prioritize, and observing how these play out under different contexts, there is much that can be learned. Some possibilities include better advocacy, teaching, and capacity strengthening of proven practice. In addition, systematic application of the approach will help to identify qualities and contexts that impact assessors can use to explain the determinants of societal and environmental impact.

RQ+ 4 CO-PRO

RQ+ 4 Co-Pro can be used by a variety of actors. These include researchers and research beneficiaries partnering in a co-production effort, but also the numerous individuals, organisations, and institutions that play stewarding roles for co-production. Here we provide four illustrative cases. This list is not exhaustive.

Case #1

Co-Producers

A co-production team uses RQ+ 4 Co-Pro from start to finish of a research project. At the outset, researchers and knowledge users apply the approach to develop and clarify a shared understanding of goals for the work. As the project progresses, the team uses the framework to assess progress, and identify areas for project improvement. When the research is completed, the team evaluates their effort against the framework components and shares successes and failures.

Case #2

Funders

A research funder launches a call for co-production research projects. To send a clear message to its community about what will be valued and assessed, the funder articulates its quality dimensions in the call. When peer-reviewers are assessing applications received, they systematically apply the framework to each application ensuring consistent and transparent reviews. Years later, the same funder revisits this portfolio of funded projects, and assesses their quality using the same framework as a post-hoc evaluation tool.

Case #3

Publishers

A journal that specializes in publishing research co-production works with its editorial board and representatives of its community to build an RQ+ 4 Co-Pro framework that represents the mission and values it wants to espouse. The journal then puts RQ+ 4 Co-Pro to work in its peer-review and editorial decision-making processes. Because it keeps a record of individual reviewer assessments, at year end the journal's editorial board commissions a meta-analysis of results against the contextual factors and quality dimensions it assessed in every paper received and published, learning much about the strengths, weaknesses, and opportunities ahead for its field.

Case #4

Universities

A university faculty decides to re-think how it supports, rewards, and teaches co-production. Using RQ+ 4 Co-Pro as an evaluative framework it begins to assess and reward faculty based on the components of quality that matter for this type of research. This sets clear and meaningful goalposts for staff who have felt co-production work was under-valued using traditional research evaluation metrics. At the same time, the faculty draws on RQ+ 4 Co-Pro as a teaching device, to share case studies and highlight critical components of co-production quality to students and staff.
